# Portable ultrasound devices for obstetric care in resource-constrained environments: mapping the landscape

**DOI:** 10.12688/gatesopenres.15088.2

**Published:** 2024-10-29

**Authors:** Bryan J. Ranger, Elizabeth Bradburn, Qingchao Chen, Micah Kim, J. Alison Noble, Aris T. Papageorghiou

**Affiliations:** 1 Department of Engineering, Boston College, Chestnut Hill, MA, 02467, USA; 2 Nuffield Department of Women's & Reproductive Health, University of Oxford, Oxford, OX3 9DU, UK; 3 Institute of Biomedical Engineering (IBME), University of Oxford, Oxford, England, UK; 4 Department of Computer Science, Boston College, Chestnut Hill, MA, 02467, USA

**Keywords:** Portable ultrasound; pregnancy; obstetrics; low resource; global health; Artificial Intelligence; Imaging; Screening

## Abstract

**Background:**

The WHO’s recommendations on antenatal care underscore the need for ultrasound assessment during pregnancy. Given that maternal and perinatal mortality remains unacceptably high in underserved regions, these guidelines are imperative for achieving better outcomes. In recent years, portable ultrasound devices have become increasingly popular in resource-constrained environments due to their cost-effectiveness, useability, and adoptability in resource-constrained settings. This desk review presents the capabilities and costs of currently available portable ultrasound devices, and is meant to serve as a resource for clinicians and researchers in the imaging community.

**Methods:**

A list of ideal technical features for portable ultrasound devices was developed in consultation with subject matter experts (SMEs). Features included image acquisition modes, cost, portability, compatibility, connectivity, data storage and security, and regulatory certification status. Information on each of the devices was collected from publicly available information, input from SMEs and/or discussions with company representatives.

**Results:**

14 devices were identified and included in this review. The output is meant to provide objective information on ideal technical features for available ultrasound systems to researchers and clinicians working in obstetric ultrasound in low-resource settings. No product endorsements are provided.

**Conclusions:**

This desk review provides an overview of the landscape of low-cost portable ultrasound probes for use in obstetrics in resource-constrained environments, and provides a description of key capabilities and costs for each. Methods could be applied to mapping the landscape of portable ultrasound devices for other clinical applications, or may be extended to reviewing other types of healthcare technologies. Further studies are recommended to evaluate portable ultrasound devices for usability and durability in global field settings.

## Introduction

Ultrasound has become an integral part of medical practice, providing timely diagnostic information. Unlike imaging using ionizing radiation such as X-ray or computer tomography (CT) scans, and at a fraction of the cost of Magnetic Resonance Imaging (MRI), ultrasound is used widely due to its cost effectiveness, portability and real-time nature. Many current clinical uses include identifying free fluid in the abdomen or pelvis (a sign of internal haemorrhage) in trauma patients; diagnosing ectopic pregnancies and ovarian cysts in gynaecology; imaging the liver, gallbladder, pancreas and appendix in general surgery; assessing of the adult heart in cardiology; and many others. However, it is in modern obstetric practice that ultrasound is the real cornerstone of care. For this reason, in this desk review we focus on obstetric ultrasound. Nevertheless, we recognize that the ultrasound devices discussed offer great benefits across the medical field.

The World Health Organization (WHO)’s *Recommendations on Antenatal Care for a Positive Pregnancy Experience* underscores the need for ultrasound assessment during pregnancy ^1^. One of the most important benefits of ultrasound is the ability to estimate gestational age (GA) which is a critical piece of information for maternity care providers to manage complications and determine the appropriate delivery of time-sensitive interventions in pregnancy ^2^. The gold standard for dating a pregnancy is to measure the crown-rump length (CRL) between 11–14 weeks. For women presenting later in pregnancy a combination of head circumference (HC) and femur length (FL) measurements can be used ^3^. Measurements of the HC, biparietal diameter (BPD), abdominal circumference (AC) and FL also provide useful information on the growth and wellbeing of the fetus. Importantly ultrasound also allows the provider to check for factors that could indicate that the pregnancy is at high risk, and benefit the mother and unborn child from increased monitoring; this includes identifying multiple pregnancies, problems with the placenta (such as placenta praevia), non-cephalic fetal presentation, increased and decreased amniotic fluid levels as well as congenital anomalies ^4^.

Ultrasound plays a critical role in antenatal care (ANC) across both high- and low-resource environments, though the levels of access and application vary between these environments. The effect of introducing antenatal ultrasound on health outcomes and systems in low-resource environments is not yet proven, but numerous sources suggest it could plausibly increase antenatal care utilization and thus lead to reduced morbidity and mortality by detecting complications and confirming fetal viability ^1, 5^. Given that maternal and perinatal mortality remain unacceptably high, particularly in underserved regions, the significance of the WHO guidelines cannot be understated ^6^. The WHO recommendations aim to improve the accuracy of GA assessments, particularly in settings with historically low ANC utilization, where inaccurate assessments can hinder the diagnosis and management of pregnancy complications.

In high-resource environments, the implementation of ultrasound services as part of ANC often benefits from widespread access to advanced technology and well-trained personnel, which supports effective use and accurate interpretation of ultrasound findings. However, despite these advantages, maintaining up-to-date training and integrating new technologies remain crucial for optimizing care. In contrast, in resource-constrained environments there is a critical lack of ultrasound equipment and skilled operators ^7^. Although the cost of devices has decreased in recent years, the lack of trained health personnel who are able to use the ultrasound device proficiently and interpret findings accurately continues to limit the quality implementation of antenatal ultrasound services in global settings ^8, 9^.

In recent years, portable ultrasound devices have become increasingly popular due to their cost-effectiveness, and usability in resource-constrained environments ^10^. Many of these new models are hand-held, mobile-based systems that are under $10,000, which is a substantially lower cost than standard full-sized clinical systems ^11^. The user-interface of such devices has been simplified and streamlined so that healthcare workers at varying levels of experience may be able to use them ^12^. As these devices are becoming more available, funding agencies and local healthcare staff will need to evaluate the strengths and weaknesses to determine which device is optimal for their intended setting.

In this desk review, we present the capabilities and costs of currently available portable ultrasound devices. This is meant to serve as a resource for clinicians, scientists, image analysis researchers, and others who are weighing the benefits and disadvantages of various ultrasound models for antenatal care in resource-constrained environments.

## Methods

A list of ideal technical features for portable ultrasound devices, with a focus on antenatal care in low resource settings, was developed by employing a user-centered approach. The list of features was established through targeted interviews with healthcare providers, technical experts, and researchers. The interviews were conducted using semi-structured questions with input from subject matter experts (SMEs) including clinicians and academic researchers who actively practice or have research programs in Ghana, India, Zambia and Kenya. The interviews were designed around key questions such as: “What ultrasound device (if any) do you use at your facilities?” “At what facility level are these devices utilized?” “How are devices used and optimized within your facility, including uses beyond obstetrics?” “What challenges do you face with the device, such as portability, durability, and training requirements?” “Do your devices have wireless connectivity, and is data upload problematic?” and “What are the top 5 features that should be prioritized for ultrasound devices?” Additionally, we explored the capacity of facilities to manage additional devices, including aspects of charging, storage, triage, staffing, and data management. After completing the interviews, we summarized our findings by analyzing common themes and constraints reported across the research sites, which allowed us to finalize a prioritized list of features. 

All features were defined *a priori*, and are outlined in Table 1. Each feature is divided into two categories: (1) ‘minimum viable product’ which represent baseline required capabilities, and (2) ‘target state’ which identify ideal attributes. The identified features consist of image acquisition modes (including extra modes for obstetrics), cost, portability, power, compatibility, connectivity, data storage and security, and certification status. These technical features are not intended to reflect differences in diagnostic accuracy of the device, but rather meant to highlight important constraints that exist in low-resource environments that should be considered carefully.

**Table 1.** Ideal technical features of portable ultrasound devices for obstetrics in resource-constrained environments.

Feature	Minimum viable product	Target state
*Image acquisition* * modes*	• Capable of scanning in M- and B-modes	• Capable of scanning in M-mode, B-mode, and color Doppler • Includes transvaginal probe option • User guidance to collect high quality images (e.g. positional sensors for automatic guidance of probe)
*Extra modes* *(for OB)*	• Capable of acquiring length (CRL, FL, BPD) and ellipse measurements (HC, AC).	• Ability to perform obstetric calculations of gestational age
*Cost (device)*	• $2000–5000	• <$1000 • Option for device to be treated as "consumable" (i.e. no need for maintenance)
*Cost (subscription)*	• Any clearly defined subscription fee	• No subscription fee
*Portability*	• Lightweight and compact size (handheld model)	• Handheld model that is compact and lightweight
*Power*	• Battery powered, with limited downtime required between uses	• Battery can last for 8-hour day, removable battery that allows for back up charge
*Compatibility* * (hardware)*	• Compatibility with mobile and tablet devices (Android)	• Compatible with mobile and tablet devices on multiple platforms, including Android and iOS
*Compatibility* *(third party software)*	• Ability for hardware to be paired with third- party software.	• Ability for hardware to be paired with third-party software through an open SDK (allowing for research and development iterations)
*Connectivity*	• USB Cable	• Wireless
*Data storage*	• Local device storage	• Cloud and mobile-based PACS
*Data security*	• Data is highly secure, encrypted either locally or on Cloud/ during data transfer	• Data is highly secure, encrypted either locally or on Cloud/ during data transfer • Facial/finger print identification for patient
*Certification status*	• FDA (510k), CE (needs to be far along in regulatory process)	• FDA (510k), CE

Candidate devices for assessment were identified through internet searches, company websites, discussions with SMEs and industry partners in the field. Only low-cost (less than $10,000) portable, mobile- or tablet-based devices were considered for this desk review. Mid- and full-size ultrasound systems were excluded given cost constraints.

Information regarding the ideal technical features of each of the devices was collected from publicly available information, input from SMEs and/or discussions with sales representatives from the respective companies. The review was conducted and verified in March 2022.

## Results

The search identified 14 portable mobile-based ultrasound systems. Ten of these employ convex probes, which are most commonly used for obstetrics due to the width of the field of view allowing improved imaging in later pregnancy; three are linear probes, which may also be useful for non-obstetric applications; while in one case a dual probe (convex and linear) has been engineered in the same device. Ten devices offered color Doppler in addition to B-mode imaging. Calculation of gestational age from fetal biometry and estimation of fetal weight were not available on all devices.

Costs ranged from $700 to $10,000 and three were available on a subscription model. Importantly, from the perspective of building new applications (including AI enabled software), no software development kit (SDK) or application program interface (API) was available for eight of the devices at the time the search was performed; while access to SDK or API was available in limited fashion or by agreement for four. In two devices, this was available by agreement with purchase.

Detailed information for each feature and for the identified products is summarized in Table 2. The output of the desk review is meant to provide objective information on available ultrasound systems for researchers and clinicians working in obstetric ultrasound in resource-constrained environments. The authors’ intention is not to make endorsements for any particular technology, and recommend that stakeholders utilize this information to make the most appropriate decisions for their given context.

**Table 2.** Portable mobile-based ultrasound systems reviewed by technical feature.

Feature	Butterfly	Philips	Clarius	GE		Konted	
	iQ+	Lumify C5-2	C3 HD	Vscan Extend	Vscan Air	C10T	Gen 4
*Probe type*	Linear	Convex	Convex	Convex	Convex & Linear	Convex	Convex
*Frequency * *range*	1–10 MHz	2–5 MHz	2–6 MHz	1.7–3.8 MHz	2–5 MHz (Curved)	3.5–5	3.5–5 MHz
*Max image* * depth*	30 cm	30 cm	40 cm	24 cm	24 cm	30.5 cm	30.5 cm
*Image modes*	B, M, CD	B, M, CD	B, M, CD	B, M, CD	B, CD	B, M, CD	B, M, CD
*Extra modes* *(for OB)*	OB calculators (Gestational age, EDD, EFW) 2D distances and ellipse measurements (BPD, HC, AC, FL)	OB calculators (Gestational age, EFW, FHR) 2D distances and ellipse measurements (BPD, HC, AC, FL)	OB calculators (Gestational age, AFI, EFW, FHR, GS) 2D distances and ellipse measurements (CRL, HC, AC, FL)	Auto Optimize Comprehensive Label Protocol Creator Screen Mirror	No OB calculators Ellipse measurements (circumference only for now), trace.	OB calculators (Gestational age, EFW) 2D distances and ellipse measurements (CRL, BPD, HC, AC, FL)	OB calculators (GA, EFW) 2D distances and ellipse measurements (CRL, BPD, HC, AC, FL)
*Image * *enhancement*	Yes, but no specific details	Yes, but no specific details	Yes, adaptive speckle reduction, edge enhancement	Yes, but no specific details	Yes, but no specific details	Yes, but no specific details	Yes, but no specific details
*Cost (device)*	$2,399	$7,000–10,000	$4,900	$4,995	$4,495	$800–1400	$700–1800
*Cost * *(subscription)*	$199–420/year	$2,388/year	N/A	N/A	N/A	N/A	N/A
*Portability*	Probe size: 6.4 × 2.2 × 1.4 in. Weight: 0.69 lbs.	Probe size: 4.5 × 1.8 × 1.2 in. Weight: 0.3 lbs.	Probe size: 6.46 × 3.07 × 1.50 in. Weight: 0.86 lbs.	Probe size: 5.08 × 1.54 × 1.1 in. Weight: 0.26 lbs.	Probe size: 5.16 × 2.52 × 1.22 in. Weight: 0.45 lbs.	Probe size: 6.18 × 2.75 × 1.18 in. Weight: 0.50 lbs.	Probe size: 6.14 × 2.36 × 0.79 in. Weight: 0.60 lbs.
*Power*	Rechargeable, 144 min. of continuous scanning	Rechargeable, 2-5 hours of continuous scanning	Rechargeable, 1 hour of continuous scanning	Rechargeable, 1 hour of continuous scanning	Rechargeable, 50 min. of continuous scanning	Rechargeable, 3 hrs. of continuous scanning	Rechargeable, 3.5 hrs. of continuous scanning
*Compatibility* * (hardware)*	Android and iOS compatible	Android and iOS compatible	Android and iOS compatible	N/A, uses GE tablet	Android and iOS compatible	Android, iOS, windows compatible	Android, iOS, windows compatible
*Compatibility* * (third party* * software)*	Limited access to SDK/API	No SDK/API	API available	No SDK/API	No SDK/API	Open with purchase	Open with purchase
*Connectivity*	USB-C or Lightning Cable	USB-C Cable	Wireless	USB cable to GE tablet	Wireless	Wireless	Wireless
*Data storage*	Cloud Storage & Smartphone- based Storage PACS (w/ subscription)	No cloud storage, Smartphone- based PACS	Cloud Storage (first 2GB Free) & Smartphone- based Storage PACS	Laptop with gateway software can access images	No cloud storage, Smartphone- based PACS	No cloud storage, Smartphone- based PACS	No cloud storage, Smartphone- based PACS
*Data security*	Standard encryption	Standard encryption	Standard encryption	Encrypted, may need additional	Standard encryption	Not available	Not available
*Certification*	FDA/CE	FDA/CE	FDA/CE	FDA/CE	FDA/CE	No FDA	No FDA
Feature	Biim	Vave	SonoQue	Healcerion	Telemed	ATL	Healson
	12-4		C3	Sonon 300C	MicrUS Pro- C60S	Cerbero	U20C
*Probe type*	Linear	Linear	Convex	Convex	Convex	Convex	Convex
*Frequency * *range*	4–12 MHz	1.5–3.5 MHz	3.5–5 MHz	3.5 MHz only	2–5 MHz	3.5–5 MHz	2.5–4.5 MHz
*Max image* * depth*	4 cm		20 cm	20 cm	31 cm	30.5 cm	24 cm
*Image modes*	B, CD	B, M, CD	B, M	B	B, M	B, M, CD	B, M
*Extra modes* *(for OB)*	No OB calculators 2D distances and circle measurements.	No OB calculators 2D distances and circle measurements.	OB Calculators (Gestational age, EFW). 2D length, area, circumference, angle, trace (GS,CRL, BPD, HC, AC, FL)	OB calculators (Gestational age) 2D distances and ellipse measurements	Distance, length, circumference, area, volume, angle, FHR	OB calculators (Gestational age, EFW) Distance, angle, are/ circumference, traced, depth. (GS, CRL, BPD, HC, AC, FL)	OB calculators (Gestational age, FHR) Perimeter and area; cross-section diagram; left/right hip angle. from (CRL, BPD, AC, BPD, FL)
*Image* * enhancement*	Yes, but no specific details	Yes, but no specific details	Yes, but no specific details	Yes, speckle Noise alleviating filter	Yes, speckle reduction, TGC control, dynamic range control	Yes, but no specific details	Yes, but no specific details
*Cost (device)*	$5,000	$1795	$1,350	$2,995	$4000-4700	Not available	Not Available
*Cost * *(subscription)*	N/A	$1,188/year	N/A	N/A	N/A	N/A	N/A
*Portability*	Probe size: 5.94 × 2.28 × 1.61 in. Weight: 0.46 lbs.	Probe size: 6.7 × 2.1 × 1.5 in. Weight: 0.75 lbs.	Probe size: 6.0 × 2.75 × 0.75 in. Weight: 0.53 lbs.	Probe size: 8.5 × 3.07 × 1.61 in. Weight: 0.66 lbs.	Probe size: 2.36 (radius) Weight: 0.44 lbs.	Probe size: 6.14 × 2.56 × 0.79 in. Weight: 0.53 lbs.	Probe size: 6.1 × 3.0 × 1 in. Weight: 0.31 lbs.
*Power*	Rechargeable, 2 hrs. of continuous scanning	Rechargeable,2 hrs. of continuous scanning	Rechargeable, 3 hrs. of continuous scanning	Rechargeable, 3 hrs. of continuous scanning	No battery, powered by USB-C	Rechargeable, 3 hrs. of continuous scanning	No information available
*Compatibility * *(hardware)*	Android and iOS compatible	Android and iOS compatible	iOS compatible	Android and iOS compatible	Android and Windows compatible	Android, iOS, Windows compatible	Android and Windows compatible
*Compatibility* * (third party* * software)*	No SDK/API	No SDK/API	No SDK/API	No SDK/API	SDK available by agreement	No SDK/API	No SDK/API
*Connectivity*	Wireless	Wireless	Wireless	Wireless	USB-C	Wireless	USB Cable
*Data storage*	No cloud storage, Smartphone- based PACS	Cloud (optional), smartphone- based PACS	Smartphone- based PACS	Smartphone- based PACS	Device storage	Device storage	Smartphone- based PACS
*Data security*	Standard encryption	Standard encryption	Standard encryption	Standard encryption	Not available	Not Available	Not Available
*Certification*	FDA/CE	FDA	FDA/CE	FDA	No FDA	No FDA	No FDA

## Conclusion/discussion

This desk review provides a review of the landscape of low-cost portable ultrasound systems for use in obstetrics in low-resource settings. Ideal technical features ( Table 1) were identified *a priori* by interviewing SMEs to guide the review of each technology. 14 low-cost portable ultrasound systems were identified, and a review of the technical features for each are presented in Table 2. The authors acknowledge that new devices are being commercialized all the time and that any review of rapidly evolving technology is of temporally finite benefit. Nevertheless, we believe that the framework we have developed can be useful for researchers and clinicians in the field to evaluate future options. Additionally, we acknowledge the limitation that information on the devices was based on that available by manufacturers. We considered that field testing was prohibitive given the number of devices available and the aforementioned rapidly developing field, and instead opted to use this review to shortlist devices for future field testing. Finally, we did not evaluate specific imaging characteristics as these can be subjective and difficult to describe in a reproducible manner.

In addition, methods for mapping the landscape, as well as the clearly defined criteria that resulted from SME discussions, may be modified for other clinical applications. For example, a comparable set of ideal technical features could be adapted to create a landscape of portable ultrasound technology for other clinical applications such as cardiac, lung, or musculoskeletal assessment. Depending on the needs of the research community, this review may also serve as a framework for mapping other types of medical technologies for applications to global healthcare.

In response to limitations related to user-dependence associated with ultrasound scanning, a research area that has seen considerable growth is artificial intelligence (AI) applied to guidance and image analysis ^13^. This has particular relevance for point-of-care ultrasound since it has the potential to guide a non-expert user to collect high quality data ^14^ and automatically interpret images to assist with clinical decision making in areas such as obstetrics ^15^. Specifically, object detection capabilities of AI are especially useful in this case, since this allows for tracking objects in real time to allow for automated image or object classification ^16^. To this end, automatic measurements of fetal crown-rump length (CRL) ^17^, biparietal diameter (BPD) and head circumference (HC) ^18^, femur length (FL) ^19^, transcerebellar diameter (TCD) ^20^, abdominal circumference (AC) ^21^, and fetal weight ^22^ are all under active investigation. In addition, advances have been made in automated gestational age estimation independent of fetal measurement ^23^, and algorithms to automatically determine fetal heart rate, placental position, fetal presentation, multiple gestation and other crucial obstetric findings are in development ^24, 25^. Given the rapid development of such AI methods to address challenges of user-dependence in global settings, utilizing ultrasound devices with the capacity to integrate AI algorithms is an important consideration for investment and future deployment. Therefore, features related to compatibility, particularly with third party software, are highlighted in Table 2.

Application Program Interface (API) and software development kit (SDK) capabilities are another important consideration when it comes to selecting a device. An API is the intermediary software that transfers information between two or more applications to communicate with each other. Modifying an ultrasound system’s API enables the extraction of specific data from that system to a mobile application. Unlike an API, which is a set of programmable libraries that come with the ultrasound system and its corresponding mobile application, an SDK is a set of tools (some of which may include APIs) that allow technical developers to create their own applications. While an API allows users to enhance the built-in application that comes with the ultrasound system, the inclusion of an SDK allows developers to build completely new applications. An SDK often comes with one or more APIs. All in all, SDKs allow for further flexibility and the ability to develop fully functioning applications from scratch using the data provided by the ultrasound system, while APIs have a more limited but still effective capability in customizing an application. Mobile based devices should have an integrable REST (representational state transfer) API capable of accessing an ultrasound system’s SDK. Ultrasound systems may rely heavily on AI models to track/detect inputs from the system’s sensor. The SDK/API associated with that particular ultrasound system will include options to modify that AI model to, for example, use object detection or perform additional computations to optimize system performance.

An important consideration for portable ultrasound devices, as well as digital health technologies more broadly, is access to the internet ^26^. In many underserved regions, internet connectivity has significantly improved, which allows for the upload of data to cloud storage or secure local servers for review or record keeping. For the portable ultrasound probes detailed in Table 2, the “Connectivity” section highlights that these devices connect to mobile platforms through either wireless or USB-C connections. Importantly, these devices do not require an active internet connection to perform a scan, and utilize on-device mechanisms for temporary storage. As a result, intermittent internet access should not pose substantial barriers to their effective use, allowing for data transfer and remote consultations when connectivity is available.

Numerous papers in the literature emphasize the need for more clinical trials and implementation studies to more adequately assess the use of portable ultrasound in low-resource settings ^11, 27, 28^. While portable ultrasound offers promising diagnostic capabilities, its real-world performance and integration into healthcare systems are not fully understood. The features provided in this review may help guide researchers to determine which portable system is most appropriate for further testing and deployment.

Numerous areas for future work are highlighted because of this desk review. In particular, the authors suggest convening a focus group of clinicians, academic clinical and AI imaging researchers, and technologists to establish trials for a selection of probes (e.g., multiple probes side-by-side at research sites of varying resources) and provide targeted feedback. As with this study, key domains for assessment must be determined in advance. It will also be essential to test probes with both male and female healthcare workers (as differences in hand sizes contribute to ergonomic performance), and with clinical healthcare workers of diverse skill levels (e.g., doctors, midwives, nurses, sonographers).

In summary, this desk review provides objective information on current available ultrasound systems. It was designed to be a resource for researchers and clinicians working in obstetric ultrasound in resource-constrained environments.

## Data Availability

No data are associated with this article.
