# Supplementary material for: Portable ultrasound devices for obstetric care in resource-constrained environments: mapping the landscape
Source: Gates Open Res. 2024 Oct 29;7:133. Originally published 2023 Dec 6. [Version 2] doi: 10.12688/gatesopenres.15088.2 (PMC11813169; doi:10.12688/gatesopenres.15088.2)
Supplement: Peer review history

# Reviewer report: Reviewer response for version 2

**DOI:** 10.21956/gatesopenres.17696.r38518

**Published:** 2024-12-24

**Authors:** Josephus van den Heuvel

**Affiliations:** 1 Utrecht University, Utrecht, The Netherlands

The authors present an overview of the available ultrasound devices for antenatal care. As a clinician and researcher of innovation in obstetric care, I am interested in the topic and the evidence provided is timely.

 It is a well written study and the authors succeeded to present the results in a comprehensive matter. The addition of most recent studies is particularly interesting for a complete overview.

 Having read the previous peer reports, this version is updated including its methods, as well as the placement of information in either introduction or discussion section. 

 I have no further comments.

Is the work clearly and accurately presented and does it cite the current literature?

Yes

If applicable, is the statistical analysis and its interpretation appropriate?

Yes

Are all the source data underlying the results available to ensure full reproducibility?

Yes

Is the study design appropriate and is the work technically sound?

Yes

Are the conclusions drawn adequately supported by the results?

Yes

Are sufficient details of methods and analysis provided to allow replication by others?

Yes

Reviewer Expertise:

digital health, obstetrics

I confirm that I have read this submission and believe that I have an appropriate level of expertise to confirm that it is of an acceptable scientific standard.

# Reviewer report: Reviewer response for version 2

**DOI:** 10.21956/gatesopenres.17696.r38815

**Published:** 2024-12-18

**Authors:** Katelyn J Rittenhouse

**Affiliations:** 1 University of North Carolina at Chapel Hill, Chapel Hill, USA

This desk review provides a detailed landscape analysis of low-cost, portable ultrasound devices tailored for obstetric care in resource-constrained settings. The authors outline ideal technical features for such devices, systematically assess 14 existing portable ultrasound systems, and provide a framework that can be extended to other clinical applications. The manuscript highlights important advancements in technology, such as artificial intelligence (AI) and integration capabilities, while also acknowledging challenges related to implementation, usability, and connectivity.

 The authors deserve credit for producing a timely and practical review. It is well-structured, addresses an urgent need, and emphasizes the importance of portable ultrasound technology in reducing maternal and perinatal morbidity and mortality in underserved regions.

 Several suggestions to improve the robustness of the study:

 1. **Standardized scoring framework**: Introducing a scoring system or a more detailed comparative matrix for device evaluation (e.g., rating devices on cost, image quality, portability, API/SDK capabilities) would allow stakeholders to interpret the results more easily. The authors could also discuss plans for future field validation studies.

 2. **Methods**: The manuscript would benefit from more detail on the SME consultation process (number of SMEs, geographic representation, professional background, etc), which would enhance transparency. 

 3. **Source Data**: While the authors provide descriptive data, they do not include full source data (i.e., interview transcripts or summaries). It would be helpful if the authors provided an appendix or supplemental material with this source information, improving reproducibility. 

 4. **Gestational Age Dating**: The authors state, "The gold standard for dating a pregnancy is to measure the crown-rump length (CRL) between 11–14 weeks" in the introduction, without a citation. I would agree that a first trimester ultrasound (<14weeks) establishing gestational age by crown-rump length is the gold standard. However, according to ISUOG and ACOG, an ultrasound performed at 6-9 weeks with a viable intrauterine pregnancy would be trusted for gestational age dating over an 11-14 week ultrasound for that pregnancy. This should be revised.

**Overall Strengths**: 

- **Timely and practical**: This review addresses an urgent need for better obstetric care tools in resource-constrained settings.
- **Well-structured framework**: The authors’ clear definition of “minimum viable” and “target state” features creates a useful framework for future assessments.
- **Forward-thinking**: The focus on AI compatibility and APIs/SDKs reflects the future direction of ultrasound technology.

**Conclusion**: 

 This manuscript is a valuable contribution to the field of obstetric care and global health technology. It presents a rigorous review of portable ultrasound devices and offers a useful framework for future evaluations. With the suggested improvements, the study will be scientifically sound, highly actionable, and of great benefit to clinicians, policymakers, and researchers working in resource-limited settings.

Is the work clearly and accurately presented and does it cite the current literature?

Yes

If applicable, is the statistical analysis and its interpretation appropriate?

Yes

Are all the source data underlying the results available to ensure full reproducibility?

No

Is the study design appropriate and is the work technically sound?

Yes

Are the conclusions drawn adequately supported by the results?

Yes

Are sufficient details of methods and analysis provided to allow replication by others?

Partly

Reviewer Expertise:

obstetric ultrasound, global health

I confirm that I have read this submission and believe that I have an appropriate level of expertise to confirm that it is of an acceptable scientific standard, however I have significant reservations, as outlined above.

# Reviewer report: Reviewer response for version 1

**DOI:** 10.21956/gatesopenres.16423.r36656

**Published:** 2024-07-19

**Authors:** Yinka Oyelese

**Affiliations:** 1 Harvard Medical School, Boston, USA

The authors present a comprehensive desk review of currently available portable ultrasound devices suitable for obstetrical use in resource-constrained environments. This is a concise and informative overview. In the introduction, the authors provide the background and rationale for the deployment of portable ultrasound devices in these settings.

 The manuscript is well-written. Notably, the categorization of device features into "minimal viable product" and "target state" is particularly effective.

 However, the introduction feels disproportionately long compared to the other sections of the manuscript. I suggest shortening the introduction and moving some of the detailed information to the discussion section. The introduction should lay the groundwork for the manuscript with a brief overview, while more in-depth discussions should be in the discussion section.

 There is a need for clarity on how SMEs were identified. Can the authors provide more information on the characteristics of these SMEs? Were any of the SMEs from environments where these devices would actually be used? This is crucial since the SMEs' perspectives shape the determination of desirable features.

 While Internet access has become more widespread, the authors should address its limitations. What are the implications if Internet access is interrupted? Additionally, it is unclear whether the authors have indicated which devices support web-based operation.

 The authors provide information on various devices, but shouldn’t OB calculators be considered a part of the "minimal viable product" for ultrasound devices used in obstetric practice? If OB calculators are not included, the devices would primarily serve to check presentation, fluid levels, and heart rates.

 The introduction mentions the use of artificial intelligence to identify and measure basic obstetric biometry, but Table 2 lacks information on automated biometric measurements. This should be addressed for completeness.

 The authors are to be commended for this valuable contribution.

Is the work clearly and accurately presented and does it cite the current literature?

Yes

If applicable, is the statistical analysis and its interpretation appropriate?

Yes

Are all the source data underlying the results available to ensure full reproducibility?

Yes

Is the study design appropriate and is the work technically sound?

Yes

Are the conclusions drawn adequately supported by the results?

Yes

Are sufficient details of methods and analysis provided to allow replication by others?

Yes

Reviewer Expertise:

Obstetric ultrasound

I confirm that I have read this submission and believe that I have an appropriate level of expertise to confirm that it is of an acceptable scientific standard, however I have significant reservations, as outlined above.

# Reviewer report: Reviewer response for version 1

**DOI:** 10.21956/gatesopenres.16423.r35862

**Published:** 2024-02-20

**Authors:** Sikolia wanyonyi

**Affiliations:** 1 Aga Khan University Hospital, Nairobi, Kenya

**Summary**

 Using a desk review approach this study aims to evaluate the technical capabilities and costs  of 14 different portable ultrasound devices, to determine their suitability for use in LMICs.

**Introduction**

 1. There  is mention of high mortality and morbidity  in LMIC. These high rates are well known to be multifactorial. Is there any association between lack of ultrasound in the facilities with the poor perinatal outcomes?

 The authors are being speculative without providing any supporting evidence to demonstrate that increased use or access to ultrasound would improve outcomes.

 2. The objective of this study was the map the landscape for portable ultrasound usage, however the authors dwell on AI use in ultrasound in a substantial proportion of their introduction. While we all can't underscore the invaluable role of AI in modern practice, the authors fails to provide a strong background to justify the study  process; or was AI part of their objective ?

**METHODS**

 1. Who defined 'ideal' technical features for LMIC? It is unfortunate that in this era of globalization we would still want to segregate even technical features in a toool that is considered important in medical practice. Why should a portable ultrasound used in LMIC be different form one used elsewhere? My suggestion is to aim to find ways in which the good portable ultrasound that is used in e.g . Europe with all ideal features would be adopted for use in LMIC  taking into account the constraints, not to define different features for the poor populations.

 2. I notice with concern that this a desk review done purely by researchers in a high income country  with aim of findings a solution for LMIC, As  much as they may have some research footing there , they may not be privy to the needs of the local  clinicians. As much as they mention they included  SMEs which included clinician  with research in LMIC, they need to demonstrate in detail the process they used  and  how they engaged the end-users to define their needs, which in my opinion should be same for all clinician regardless of where they practice.

 3. The study would have been more informative if they  had just evaluated the different aspects of the probable ultrasound devises without prescribing features for a certain region.

**REFERENCES**

 A lot of work has been done on the constraints  and  ultrasound needs in LMICs by local authors but these are not cited in this study.  In my assessment this may mean the authors did not do sufficient relevant literature review on works already done in LMICs on the subject.

Is the work clearly and accurately presented and does it cite the current literature?

Yes

If applicable, is the statistical analysis and its interpretation appropriate?

Not applicable

Are all the source data underlying the results available to ensure full reproducibility?

No source data required

Is the study design appropriate and is the work technically sound?

Partly

Are the conclusions drawn adequately supported by the results?

Yes

Are sufficient details of methods and analysis provided to allow replication by others?

No

Reviewer Expertise:

Fetal growth, prenatal diagnosis and obstetric ultrasound

I confirm that I have read this submission and believe that I have an appropriate level of expertise to state that I do not consider it to be of an acceptable scientific standard, for reasons outlined above.
